# Correlation of dietary flavonoid intake with chronic bronchitis, emphysema, and asthma in U.S. adults: A large, national, cross-sectional study

**DOI:** 10.1371/journal.pone.0309310

**Published:** 2024-10-21

**Authors:** Mengshi Sun, Qin Ding

**Affiliations:** 1 Department of Gynecological Tumor Surgery, Hubei Cancer Hospital, Tongji Medical College, Huazhong University of Science and Technology, Wuhan, China; 2 Department of Gastroenterology, Hubei Cancer Hospital, Tongji Medical College, Huazhong University of Science and Technology, Wuhan, China; American University of Madaba, JORDAN

## Abstract

**Objective:**

To explore the relationship between dietary flavonoids and bronchitis, emphysema and asthma.

**Method:**

A total of 11743 United States adults were extracted from the National Health and Nutrition Examination Survey (NHANES) in 2007–2008, 2009–2010 and 2017–2018. Of these, 47.7% were male and 52.3% female. Dietary flavonoid intake assessed using FDNNS and 24-hour dietary recall data. Inclusion of demographics (gender, age, education, family income), behavioral factors (BMI, smoking, drinking status, diet), chronic disease information (diabetes, hypertension) as covariates to eliminate confounding. Stepwise logistic regression was used to analyze the association between total dietary flavonoid intake and the risk of chronic respiratory disease. Weighted quantile sum regression (WQS) was used to analyze the association between 29 dietary flavonoids and the risk of chronic respiratory disease. Restricted cubic spline was used to analyze the dose-response relationship between dietary flavonoid intake and risk of chronic respiratory disease.

**Results:**

Stepwise logistic regression results showed that higher flavonoid intake in men was associated with a lower risk of CB and asthma (OR of CB: 0.55(0.31–0.97); OR of asthma: 0.72(0.52–0.99)), and WQS results showed a mixed health effect for total flavonoids and chronic respiratory tract in response to the 29 flavonoid fractions (OR of asthma: 0.97(0.94–0.99); OR of emphysema: 0.95(0.90–0.99)). Glycitein had the highest health contribution of 26.2% for emphysema; Eriodictyol had the highest health contribution of 32.13% for asthma, respectively. The RCS showed a dose-response relationship between flavonoids and respiratory tract health. The maximum dose for ingesting flavonoids to gain respiratory health benefits is 1500 mg/d.

**Conclusion:**

Higher dietary flavonoid intake was associated with lower chronic respiratory risk in adult U.S. men. Also 29 dietary flavonoid components have an overall health effect on respiratory health. Glycitein and Eriodictyol may have potential health effects on the respiratory system. 1500 mg/day may be the Tolerable Upper Intake Level of dietary flavonoids for respiratory health in U.S. adults.

## Introduction

Chronic respiratory diseases represent a significant global public health issue, characterized not only by a substantial disease burden but also by a reduction in healthy life expectancy and an increase in premature mortality [1, 2]. Conditions such as asthma, chronic bronchitis, and emphysema are widespread in the United States. Asthma, in particular, affects approximately 8% of the adult population in the U.S. [3]. Chronic bronchitis and emphysema are recognized as distinct phenotypes within the spectrum of chronic obstructive pulmonary disease (COPD). COPD significantly impacts over 15 million individuals across the nation, ranking as the third leading cause of death both in the United States and on a global scale [4].

In the realm of chronic respiratory diseases, aside from genetic predispositions and allergic reactions, the primary etiological factors are infections or exposure to noxious environmental agents. These agents exert detrimental effects on the pulmonary system and the respiratory tract, leading to a chronic pathological state [5]. Prolonged inflammatory stress within the respiratory tract can result in tissue damage, ultimately leading to the formation of organic lesions and the progression to chronic respiratory diseases. In the context of prevention and management of these conditions, current clinical guidelines underscore the lack of specific pharmaceutical treatments [5–7]. Instead, they emphasize the significance of adopting a healthy lifestyle to improve the overall well-being and quality of life for affected individuals [8, 9].

In recent decades, there has been a growing body of research on the relationship between diet and chronic diseases [10–12]. Flavonoids, a class of naturally occurring bioactive compounds present in plant-based foods, exhibit a range of beneficial properties, including immune modulation, anti-inflammatory effects, and antimicrobial activity [13, 14]. These compounds are known to modulate the release of chemical mediators and cytokines, attenuate the synthesis of pro-inflammatory substances, and function as potent antioxidants. Empirical research has demonstrated a significant correlation between flavonoid consumption and the mitigation of chronic conditions such as diabetes, inflammatory disorders, sleep disturbances, and cardiovascular diseases [15–17]. However, most of the research conducted on flavonoids and chronic respiratory diseases has focused on pharmacology, cellular, and animal models, while larger population-based epidemiological studies remain limited [18, 19]. This study utilizes the National Health and Nutrition Examination Survey (NHANES) database, a comprehensive and extensive cross-sectional survey in the United States, to investigate the correlation between flavonoid intake and respiratory diseases. Considering the ubiquity of flavonoids in everyday diets and the elevated incidence of respiratory conditions such as asthma, chronic bronchitis, and emphysema within the U.S. populace, this investigation holds profound implications for public health.

## Materials and methods

### Study sample

**Fig 1:**
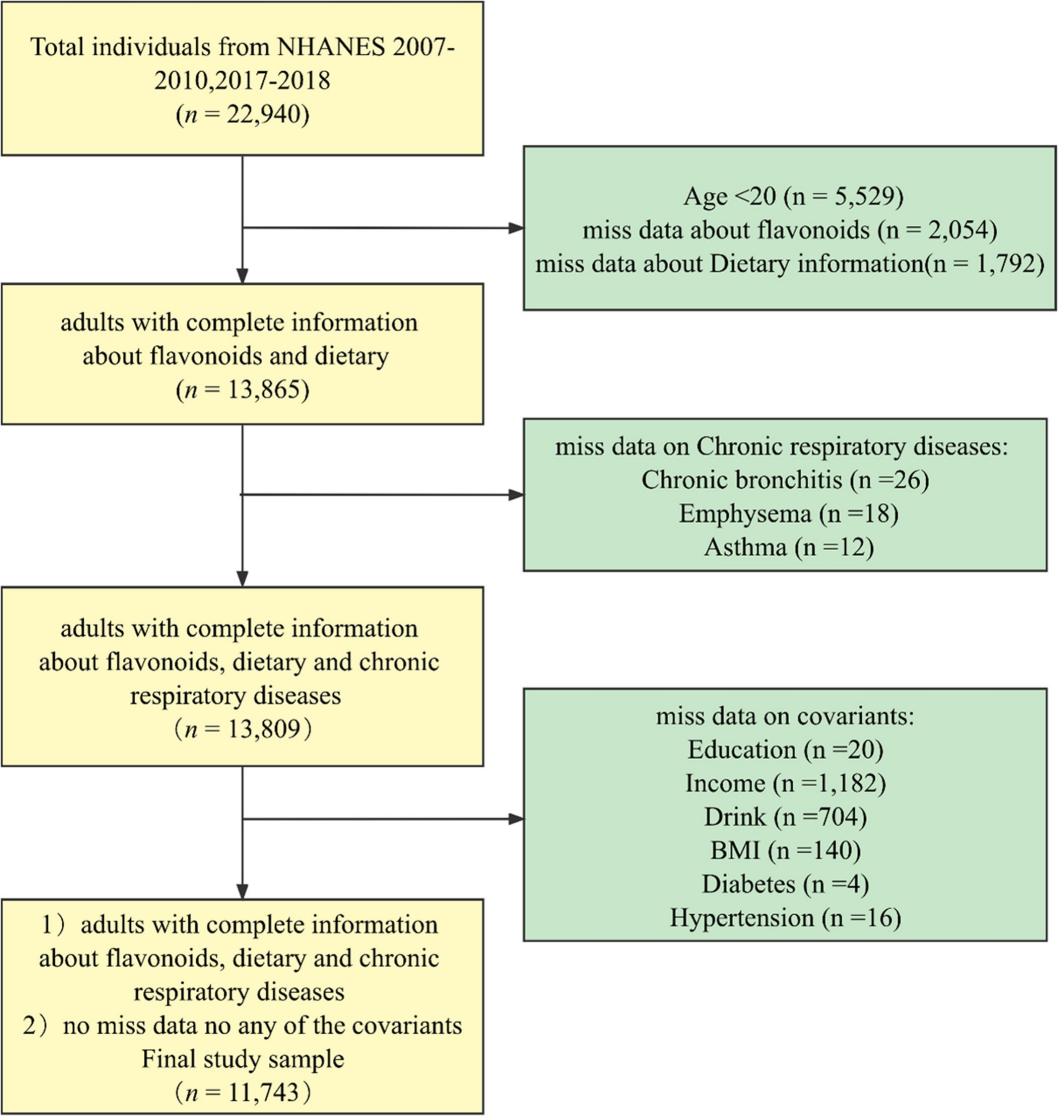
Flowchart of the study population.

The NHANES database, conducted regularly by the National Center for Health Statistics (NCHS) under the Centers for Disease Control and Prevention (CDC), is a cross-sectional study that examines the nutritional intake and health-related conditions of populations in the United States [20]. This survey employs a complex, multistage sampling design to ensure the survey results are representative of the diverse populations throughout the country. Due to limitations in obtaining data on flavonoid intake, this study incorporated data from three NHANES survey cycles: 2007–2008, 2009–2010, and 2017–2018. The sample utilized in this study consisted of data encompassing complete demographics (gender, age, education, family income), behavioral factors (BMI, smoking, drinking status, diet), chronic disease information (diabetes, hypertension), and flavonoid intake data. With a final sample size of n = 11,743, the data was weighted to accurately represent the non-institutionalized adult U.S. population, estimated at 150 million individuals. Refer to **Fig 1** for a visual representation of the process utilized.

### Flavonoids and diet quality

#### Flavonoids

The calculation of dietary flavonoid intake in this study utilized the USDA Food and Nutrient Database for Dietary Studies (FNDDS) [21]. The amount of different flavonoids in each diet is estimated from the What We Eat in America (WWEIA) [22]. Based on the 2-day, 24-hour dietary recall survey data and WWIE documentation, respondents’ dietary intake data were calculated as total dietary flavonoid intake and intake of 29 flavonoid components. The29 flavonoid components, namely, Daidzein, Eisenstein, Glycitein, Cyanidin, Petunidin, Delphinidin, Malvidin, Pelargonidin, Peonidin, Catechin, Epigallocatechin, Epicatechin, Epicatechin 3-gallate, Epigallocatechin 3-gallate, Theaflavin, Thearubigins, Eriodictyol, Hesperetin, Naringenin, Apigenin, Luteolin, Isorhamnetin, Kaempferol, Myricetin, Quercetin, Theaflavin-3,3’-digallate, Theaflavin-3’-gallate, Theaflavin-3-gallate, and Gallocatechin. To conduct weighted Scott-Rao chi-square tests and weighted logistic regressions, the total dietary flavonoid intake is categorized into four groups using quartiles: Q1 (lowest intake group, reference group), Q2, Q3, and Q4 (highest intake group) [23].

#### Diet quality

The purpose of HEI-2015 is to evaluate adherence to the Dietary Guidelines for Americans (DGA), which consists of 13 components [24]. These components include the adequacy components such as total vegetables, greens and beans, total fruits, whole fruits, whole grains, dairy, total protein foods, seafood, and plant proteins, as well as the moderation components such as sodium, refined grains, saturated fats, and added sugars. Each component is assigned different weights and has different maximum scores. HEI-2015 scores range from 0 to 100, with higher scores indicating better diet quality. To conduct weighted Scott-Rao chi-square tests and weighted logistic regressions, the HEI-2015 score is also categorized into four groups using quartiles: Q1 (lowest diet quality, reference group), Q2, Q3, and Q4 (highest diet quality) [25].

### Outcome variable of chronic respiratory diseases

Three chronic respiratory diseases, namely chronic bronchitis, emphysema, and asthma, were chosen as the outcome measures for this study.

#### Chronic bronchitis

All subjects were asked “Has a doctor or other health professional ever told you that you had chronic bronchitis?” and then categorized into non-patients (reference group) and patients according to the answers [26].

#### Emphysema

All subjects were asked “Has a doctor or other health professional ever told you that you had emphysema?” and then categorized into non-patients (reference group) and patients according to the answers [26].

#### Asthma

All subjects were asked “Has a doctor or other health professional ever told you that you had asthma:?” and then categorized into non-patients (reference group) and patients according to the answers [26].

### Covariates

#### Gender

Gender was classified as female (reference group) and male.

#### Age

The age group was categorized into 20–39 years group (reference group), 40–59 years group, 60–79 years group, 80+ years group [27].

#### Race

The race group was categorized into Mexican American group (reference group), Other Hispanic group, Non-Hispanic White group, Non-Hispanic Black group, Other Race group [28].

#### Education

Education level was categorized as less than high school (reference group), high school graduate/GED, some college/AA degree, and college graduate or more [29].

#### Family income

Family income was categorized as ≤130% (reference group), >130% to 350%, and >350% by the ratio of family income to poverty (FPL) [30].

#### BMI status

Body mass index was calculated from measured height and weight as weight/height2 (kg/m2), then categorized into normal Weight (reference group, ≥18.5 to 24.9), underweight (<18.5), overweight (≥25 to 29.9), obese (≥30) [31].

#### Smoking status

Smoking behavior was assessed using the "Smoking: Cigarette Use" questionnaire. Respondents were asked if they had smoked a minimum of 100 cigarettes throughout their lifetime. If the response was negative, they were categorized as never smokers (reference group). If the respondent had smoked at least 100 cigarettes in their lifetime and currently smokes at the time of questionnaire completion, they are classified as current smokers. Respondents who had smoked 100 cigarettes in their lifetime but had quit smoking at the time of questionnaire completion were classified as former smokers [32].

#### Drinking status

Drinking behavior was measured by the "Alcohol Use" questionnaire. The respondent was asked Ever had a drink of any kind of alcohol, and then categorized into no alcohol consumption (reference group) and alcohol consumption [33].

#### Hypertension

All participants were asked, “Ever been told by a doctor or other health professional that you had hypertension?”, and they were classified into two groups: non-patients (reference group) and patients [34].

#### Diabetes

During the NHANES home interviews, all participants were asked, "Ever been diagnosed with diabetes or sugar diabetes by a doctor or other health professional?" They were then classified into two groups: non-patients (reference group) and patients [35].

### Ethics declarations

NHANES was approved by the Institutional Review Board of the Centers for Disease Control and Prevention and written informed consent was obtained from all participants.

### Statistical analysis

The baseline characteristics of different groups were tested using the Scott-Rao chi-square tests and T-test. Weighted steps forward (likelihood ratio) binary logistic regression models were employed to evaluate the association between flavonoid intake and chronic bronchitis, emphysema, and asthma. Model 1 corrects for demographic sociological variables (gender, age, race, education, household income). Model 2 further corrects for behavioral variables (BMI, smoking, alcohol consumption, diet quality). Model 3 further corrects for chronic disease covariates (diabetes, hypertension). Restricted cubic splines (RCS) were utilized to examine the dose-response relationship between flavonoid intake and these respiratory conditions. The effects of combined exposure to 29 flavonoid components were assessed using the weighted quartile sum (WQS) regression model. This model calculates a weighted regression index representing the overall health impact of all 29 flavonoid components. The data for the WQS models were randomly divided into two sets, with 40% as the training set and 60% as the validation set. To validate the degree of WQS model fit, a receiver operating characteristic curve (ROC) was employed. All measures were accompanied by 95% confidence intervals (CIs).

The NHANES sample weights were employed in the modeling analysis to obtain unbiased estimates of the U.S. population. To address errors resulting from complex sampling weights, the CDC-recommended Taylor linearization weighting model was utilized.

All statistical tests were two-sided, and significance was considered at *P* < 0.05. WQS and ROC were performed with the R (version 4.1.0). RCS was implemented with the R package “rms (version 6.3–0). WQS was implemented with the R package “gWQS” (version 3.0.4).

## Results

### Characteristics of the baseline population

After excluding missing values for variables, a total of 11,743 subjects were included in the analysis. These subjects were representative of 170 million non-institutionalized U.S. adults. The proportion of males in the study population was 47.7% and females 52.3%. There was no difference in intake of dietary flavonoids between genders (male: 226.19 mg/d, female: 227.25 mg/d) **Table 1** provides baseline information on the sample, showing gender differences in the prevalence of chronic bronchitis and asthma disease, but no gender differences in total flavonoid intake. And from the baseline data, it also can be seen that compared to women, men are more likely to be middle-aged, highly educated, have higher incomes, be overweight, smoke and drink, have lower dietary quality, and have a lower prevalence of chronic respiratory diseases.

**Table 1:** The baseline population characteristics among U.S. adults aged 20 years or older.

Characteristics	Total	male	female	P-Value
	11743(100%)	5671(47.7%)	6072(52.3%)	<0.05
**age (mean (SD))**	47.84(16.67)	47.24(16.46)	48.38(16.84)	<0.05
**Age Group No. (Weighted%)**				<0.05
20–39 y	3670(35.2%)	1739(36.3%)	1931(34.2%)	
40–59 y	3849(38.2%)	1830(38.5%)	2019(37.9%)	
60–79 y	3501(22.9%)	1749(22.2%)	1752(23.6%)	
80+ y	723(3.7%)	353(3.1%)	370(4.4%)	
**Race No. (Weighted%)**				<0.05
Mexican American	1781(7.6%)	849(8.1%)	932(7.1%)	
Other Hispanic	1095(4.9%)	498(4.9%)	597(4.8%)	
Non-Hispanic White	5580(70.4%)	2746(70.5%)	2834(70.3%)	
Non-Hispanic Black	2360(10.5%)	1124(9.7%)	1236(11.3%)	
Other Race	927(6.7%)	454(6.8%)	473(6.6%)	
**Education No. (Weighted%)**				<0.05
< High School	2789(14.7%)	1414(15.3%)	1375(14.2%)	
High school /GED	2794(24.8%)	1398(25.9%)	1396(23.7%)	
College/AA degree	3515(30.5%)	1561(29%)	1954(31.9%)	
College or above	2645(30%)	1298(29.8%)	1347(30.2%)	
**Family Income No. (Weighted%)**				<0.05
0∼130 FPL	3450(19.1%)	1537(17%)	1913(21%)	
>130 ∼350 FPL	4606(35.6%)	2249(35.6%)	2357(35.5%)	
>350 FPL	3687(45.3%)	1885(47.3%)	1802(43.5%)	
**BMI (mean (SD))**	29(6.9)	29(6.05)	29(7.6)	0.49
**BMI No. (Weighted%)**				<0.05
Underweight	167(1.5%)	66(1%)	101(1.9%)	
Normal Weight	2977(27.2%)	1350(23.2%)	1627(30.8%)	
Overweight	3902(32.6%)	2127(37.2%)	1775(28.3%)	
Obese	4697(38.8%)	2128(38.6%)	2569(38.9%)	
**Drink Level No. (Weighted%)**				<0.05
No alcohol consumption	2625(17.9%)	759(11.2%)	1866(23.9%)	
Alcohol consumption	9118(82.1%)	4912(88.8%)	4206(76.1%)	
**Smoke Status No. (Weighted%)**				<0.05
Never Smoker	6353(55.5%)	2573(48.5%)	3780(61.9%)	
Former Smoker	2362(18.8%)	1305(20.7%)	1057(17.1%)	
Current Smoker	3028(25.7%)	1793(30.9%)	1235(21%)	
**Diabetes No. (Weighted%)**				0.06
No	10205(90.4%)	4866(89.7%)	5339(91.2%)	
Yes	1538(9.6%)	805(10.3%)	733(8.8%)	
**Hypertension No. (Weighted%)**				0.25
No	7402(68.3%)	3562(67.6%)	3840(69%)	
Yes	4341(31.7%)	2109(32.4%)	2232(31%)	
**HEI Category No. (Weighted%)**				<0.05
Q1	2936(25.9%)	1608(29.2%)	1328(22.9%)	
Q2	2935(25.2%)	1438(26.4%)	1497(24.2%)	
Q3	2936(24.3%)	1398(23.2%)	1538(25.3%)	
Q4	2936(24.6%)	1227(21.3%)	1709(27.6%)	
**flavonoid Category No. (Weighted%)**				0.32
Q1	2932(23.4%)	1405(23.6%)	1527(23.2%)	
Q2	2937(23.7%)	1447(23.9%)	1490(23.6%)	
Q3	2934(24.9%)	1422(25.5%)	1512(24.3%)	
Q4	2936(28%)	1394(27%)	1542(29%)	
**flavonoid (mean (SD))**	226.75 (393.07)	226.19 (393.29)	227.25 (392.91)	0.92
**chronic bronchitis No. (Weighted%)**				<0.05
no	11008(94.2%)	5387(95.6%)	5621(92.9%)	
yes	735(5.8%)	284(4.4%)	451(7.1%)	
**emphysema No. (Weighted%)**				0.37
no	11471(98.2%)	5504(98.1%)	5967(98.3%)	
yes	272(1.8%)	167(1.9%)	105(1.7%)	
**asthma No. (Weighted%)**				<0.05
no	10050(85.8%)	4962(87.9%)	5088(83.9%)	
yes	1693(14.2%)	709(12.1%)	984(16.1%)	

### Sex differences in the relationship between flavonoid intake and risk of chronic respiratory disease

A multifactorial, stepwise logistic regression model was utilized to investigate the correlation between flavonoid consumption and risk of chronic respiratory diseases. Continuously correcting for additional covariates, logistic regression results showed no correlation between flavonoid intake and risk of chronic respiratory disease **(Table 2)**.

**Table 2:** Relationship between total flavonoid intake and three chronic respiratory diseases among U.S. adults aged 20 years or older.

Disease	variables(ref)	OR (95%CI)		
		model 1	model 2	model 3
Chronic bronchitis	flavonoid(Q1)			
	Q2	0.81 (0.60,1.10)	0.95 (0.71,1.27)	0.96 (0.71,1.28)
	Q3	0.78 (0.57,1.08)	0.97 (0.68,1.39)	1.00 (0.70,1.44)
	Q4	0.92 (0.65,1.29)	1.07 (0.78,1.47)	1.09 (0.79,1.51)
Emphysema	flavonoid(Q1)			
	Q2	0.56 (0.38,0.82)	0.71 (0.47,1.07)	0.72 (0.48,1.1)
	Q3	0.56 (0.36,0.86)	0.82 (0.49,1.37)	0.84 (0.5,1.41)
	Q4	0.79 (0.53,1.17)	1.05 (0.70,1.56)	1.08 (0.72,1.63)
Asthma	flavonoid(Q1)			
	Q2	0.81 (0.68,0.97)	0.88 (0.72,1.08)	0.88 (0.72,1.08)
	Q3	0.95 (0.75,1.20)	1.06 (0.83,1.35)	1.07 (0.84,1.36)
	Q4	0.86 (0.73,1.01)	0.93 (0.78,1.09)	0.93 (0.79,1.10)

Model 1 = Gender + Age group + Race + Education + Famliy income.

Model 2 = Model 1 + BMI + Drink Level + Smoke Status + HEI Category.

Model 3 = Model 2 + Diabetes + Hypertension.

Compared to the female population, however, the correlation between flavonoid intake and low risk of chronic respiratory disease was more pronounced in the male population. The results showed that higher total flavonoid intake was significantly associated with a lower risk of chronic bronchitis (OR: 0.55, 95% CI: 0.31–0.97, P<0.05) and asthma (OR: 0.72, 95% CI: 0.52–0.99, P<0.05) among adult US males (**Table 3**).

**Table 3:** Association between total flavonoid intake and three chronic respiratory diseases in gender subgroup of U.S. adults aged 20 years and older.

Disease outcome	Male		Female	
	OR(95%CI)	*P-value*	OR(95%CI)	*P-value*
**Chronic bronchitis**				
Model 1	0.69 (0.44,1.07)	0.11	1.17 (0.83,1.66)	0.37
Model 2	0.55 (0.31,0.97)	0.04	1.39 (0.92,2.11)	0.14
Model 3	0.83 (0.52,1.34)	0.46	1.27 (0.88,1.85)	0.22
**Emphysema**				
Model 1	0.66 (0.32,1.33)	0.25	0.74 (0.41,1.34)	0.34
Model 2	0.61 (0.31,1.19)	0.16	1.10 (0.44,2.78)	0.84
Model 3	0.73 (0.38,1.37)	0.33	1.46 (0.84,2.54)	0.20
**Asthma**				
Model 1	0.79 (0.58,1.07)	0.14	0.96 (0.73,1.25)	0.74
Model 2	0.92 (0.67,1.27)	0.62	1.18 (0.86,1.62)	0.31
Model 3	0.72 (0.52,0.99)	0.04	1.09 (0.86,1.37)	0.49

Model 1 = Age group + Race + Education + Famliy income.

Model 2 = Model 1 + BMI + Drink Level + Smoke Status + HEI Category.

Model 3 = Model 2 + Diabetes + Hypertension.

### U-shaped dose-response relationship between flavonoid intake and risk of chronic respiratory diseases

The restricted cubic spline (RCS) model was employed to investigate the dose-response relationship between total flavonoid intake and the risk of chronic respiratory diseases, treating total flavonoid intake as a continuous variable.

**Fig 2:**
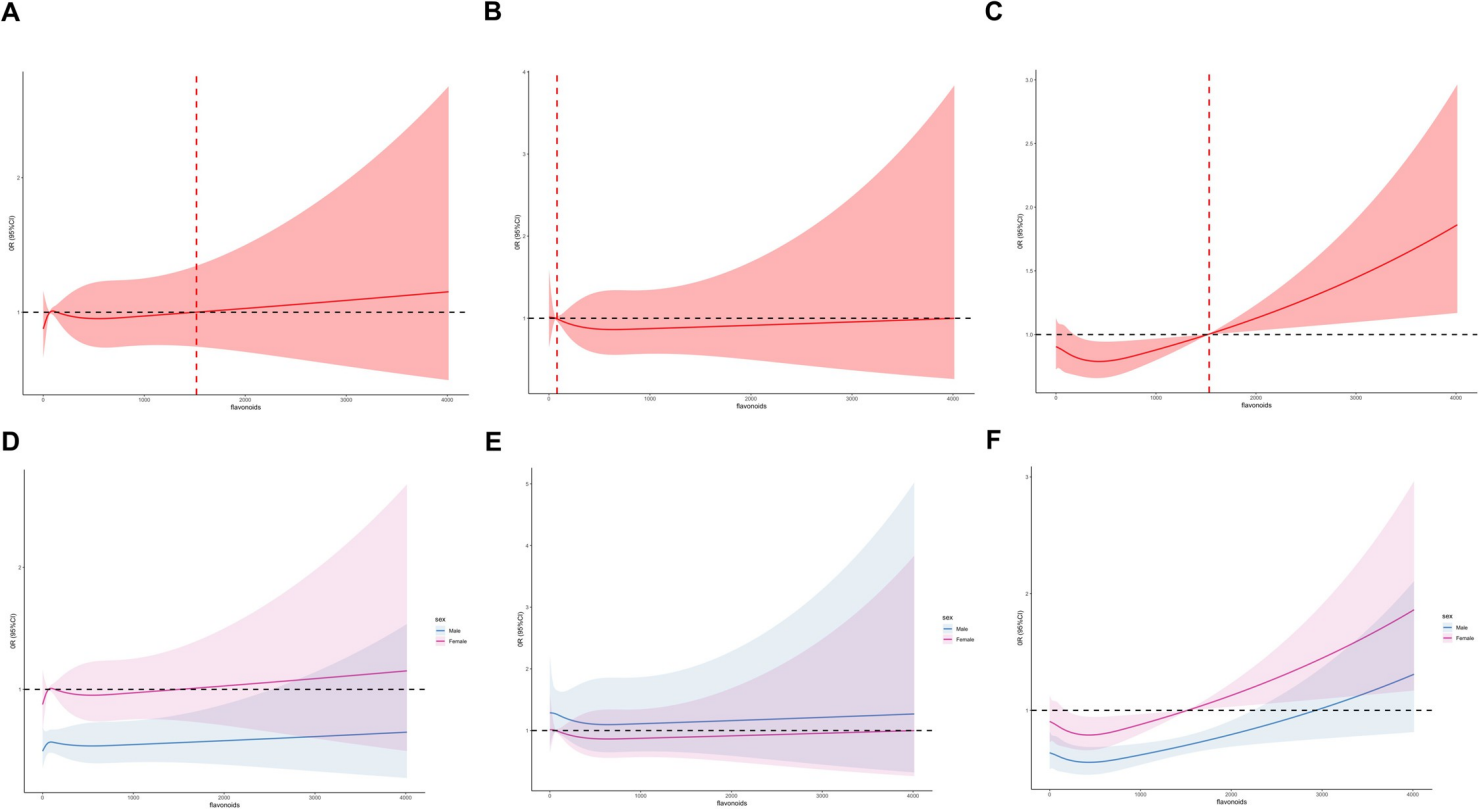
Dose–response association between flavonoid (in continues) and chronic bronchitis/emphysema/asthma using restricted cubic splines(RCS). The whole population models of chronic bronchitis (A) and models of gender stratification (D). The whole population models of emphysema (B) and models of gender stratification (E). The whole population models of asthma (C) and models of gender stratification (F) The whole population models were adjusted for gender, age group, race, education, family income, BMI, smoking status, drinking, HEI, hypertension, diabetes. The models of gender stratification were adjusted for age group, race, education, family income, BMI, smoking status, drinking, HEI, hypertension, diabetes.

**Fig 2** shows that there was no significant dose-response relationship between the flavonoid intake and chronic bronchitis / emphysema among U.S. adults. However, a significant dose-response relationship was observed between total flavonoid intake and asthma. This relationship was found to be nonlinear, indicating that increased flavonoid intake could potentially increase the risk of asthma after reaching a threshold of 1500 mg/d. These findings suggest a possible U-shaped dose-response relationship between total flavonoid intake and the risk of asthma. In the male subgroup, a dose-response relationship was observed between total flavonoid intake and chronic bronchitis/asthma, and this relationship exhibited a significant protective effect, consistent with the results of weighted logistic regression.

### Mixed health effects of 29 flavonoid components on chronic respiratory diseases

The WQS model was used to further analyse the effects of different types of flavonoids on respiratory health effects. Dietary total flavonoid intake was classified into 29 flavonoid components in the WQS model. The outcomes revealed significant mixed health effects associated with total flavonoid intake for asthma (OR: 0.97, 95% CI: 0.94–0.99) and emphysema (OR: 0.95, 95% CI: 0.90–0.99). Additionally, there was a tendency for a mixed effect between total flavonoids and chronic bronchitis, but it did not reach statistical significance (OR: 0.85, 95% CI: 0.70–1.03) (**Table 4**).

**Table 4:** Relationship between WQS regression index and chronic bronchitis / emphysema / asthma among adults aged 20 years or older.

Disease outcome	OR(95%CI)	*P-value*
**Chronic bronchitis**		
Model 1	0.93(0.89,0.97)	0.00
Model 2	0.98(0.94,1.02)	0.21
Model 3	0.85(0.70,1.03)	0.09
**Emphysema**		
Model 1	0.82(0.75,0.90)	0.00
Model 2	0.92(0.82,0.99)	0.03
Model 3	0.95(0.90,0.99)	0.04
**Asthma**		
Model 1	0.95(0.93,0.98)	0.00
Model 2	0.97(0.94,0.99)	0.02
Model 3	0.97(0.94,0.99)	0.04

Model 1 = Gender + Age group + Race + Education + Famliy income.

Model 2 = Model 1 + BMI + Drink Level + Smoke Status + HEI Category.

Model 3 = Model 2 + Diabetes + Hypertension.

**Fig 3:**
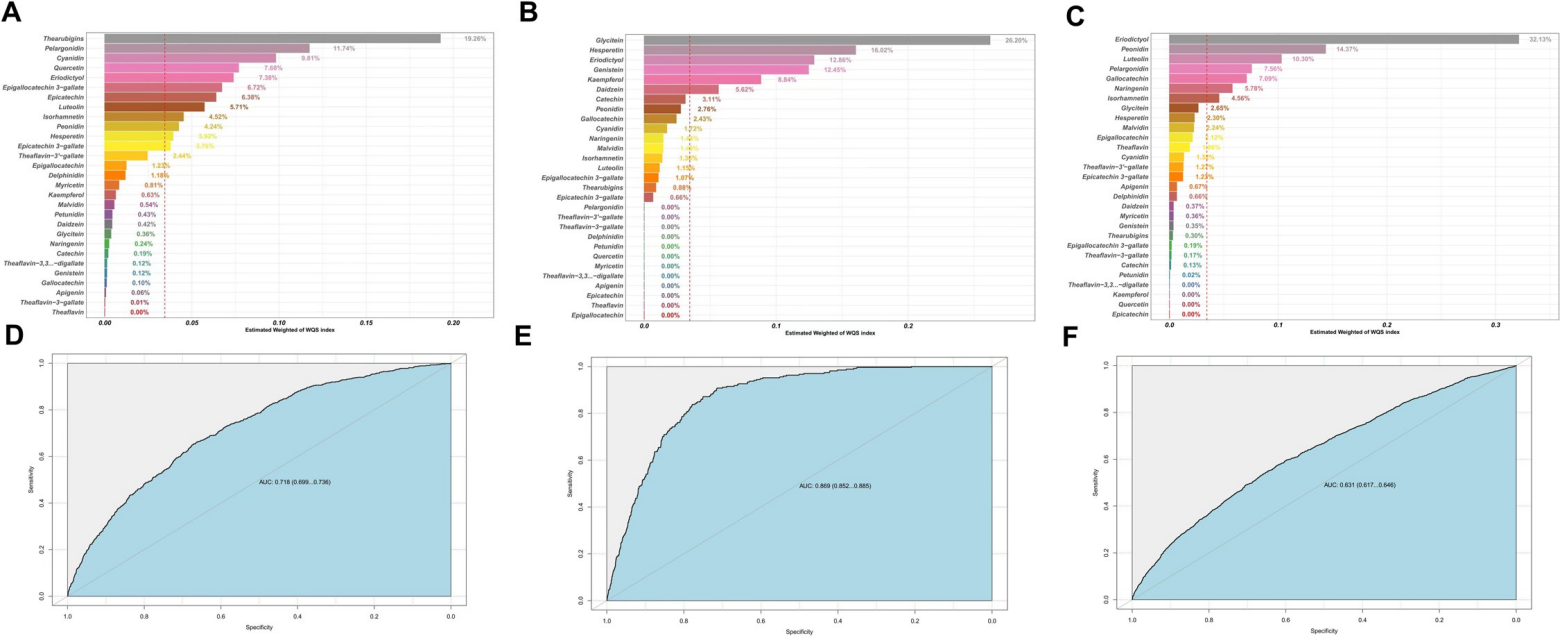
WQS model regression index weights for chronic bronchitis (A) and the AUCs of the WQS models (D). WQS model regression index weights for emphysema (B) and the AUCs of the WQS models (E). WQS model regression index weights for asthma (C) and the AUCs of the WQS models (F). WQS models were adjusted for gender, age group, race, education, family income, BMI, smoking status, drinking, HEI, hypertension, diabetes.

**Fig 3** presents the results of the health contributions of different flavonoid components. Glycitein, Hesperetin, Eriodictyol, and Genistein were identified as the top contributors, accounting for 26.2%, 16.02%, 12.86%, and 12.45% respectively, to the health effects of emphysema among the 29 flavonoid components. Similarly, Eriodictyol, Peonidin, and Luteolin were the major contributors, representing 32.13%, 14.37%, and 10.30% respectively, to the health effects of asthma among the total flavonoids. In the non-significant health effects trend observed in the WQS-chronic bronchitis model, Thearubigins, Pelargonidin, and Cyanidin emerged as the leading contributors, accounting for 19.26%, 11.74%, and 9.81% respectively.

## Discussion

Our study encompassed 11,743 participants, carefully selected to be demographically representative of approximately 150 million non-institutionalized adults in U.S. through the application of appropriate weighting techniques. Utilizing weighted logistic regression, RCS and WQS model analysis, our findings suggest that a higher intake of total flavonoids in the general population is associated with a lower risk of developing the three chronic respiratory diseases. This relationship was more significant in males. Among males, we observed a stronger association between higher total flavonoid intake and a lower risk of chronic bronchitis and asthma. The results from the restricted cubic spline analysis further supported this, demonstrating a significant dose-response relationship between total flavonoid intake and chronic bronchitis as well as asthma in males. In addition, our findings suggest that there is a U-shaped dose-response relationship between flavonoid intake and asthma in the entire study population, and that 1500 mg/day may be the tolerable upper intake levels of dietary flavonoids for adults in the United States. The results of the weighted quantile sum (WQS) modeling indicated that total flavonoid intake, consisting of 29 flavonoid compounds, exhibited a beneficial mixed effect on emphysema and asthma within the total population. Among the various flavonoid components, glycitein, hesperetin, eriodictyol, and genistein contributed the most to the health effects on emphysema, accounting for 26.2%, 16.02%, 12.86%, and 12.45% respectively. Similarly, eriodictyol, peonidin, and luteolin were the top contributing components for the health effects on asthma within the total flavonoid intake, making up 32.13%, 14.37%, and 10.30% respectively.

Oxidative stress is one of the most common triggers for initiating and exacerbating respiratory diseases. Flavonoids are well known for their anti-inflammatory and antioxidant properties, and there is a wealth of experimental evidence linking flavonoids to anti-inflammatory, anti-aging and immunomodulatory effects. Several flavonoids effectively exert anti-inflammatory effects by blocking NF-κB and NLRP3 inflammatory vesicles, inhibiting the production of inflammatory cytokines such as IL-1β, IL-2, IL-6, and TNF-α, downregulating chemokines, and reducing reactive nitrogen species and reactive oxygen species (ROS). The flavonoids with the highest efficacy in terms of inflammation and immune response modulation are apigenin, quercetin, and epigallocatechin-3-gallate (EGCG) [36]. In the realm of epidemiological research, a scarcity of population-based studies exists that delve into the correlation between flavonoid intake and respiratory diseases, with the extant findings exhibiting a notable lack of uniformity. Numerous studies grounded in population-based methodologies have posited that an elevated intake of foods abundant in flavonoids, encompassing a spectrum of items such as fruits, vegetables, wine, and tea, is associated with a diminished risk of developing respiratory ailments [37–39]. However, this association has not been replicated in other studies [40]. Tabak et al. reported that a higher intake of catechins among Dutch adults was associated with a reduced risk of chronic obstructive pulmonary disease (COPD) and asthma [41]. Conversely, Garcia et al. found no evidence linking the three subclasses of flavonoids to asthma or chronic bronchitis (CB) in a population-based case-control study in London [42]. Furthermore, a recent clinical trial involving adults and children with controlled asthma showed no significant association between the supplemental use of soy isoflavones and lung function [43]. The findings of our study provide additional evidence from population-based epidemiological studies, suggesting an association between dietary flavonoid intake and a decreased risk of three chronic respiratory diseases among adults in the United States.

Moreover, our study findings demonstrate gender disparities in the association between flavonoid intake and the risk of chronic respiratory diseases within the US population, with men exhibiting greater sensitivity. The relevant study found that the prevalence of asthma is significantly higher among males than females in the U.S. [44]. In a study of dietary flavonoid intake and risk of metabolic syndrome, the health effects of flavonoids were also found to be more significant in males [45]. The likely reason for this is that the prevalence of disease is more significant in men, so the health benefits from flavonoids would also be more significant. Additionally, it is plausible that phytoestrogens present in flavonoids contribute to a bi-directional regulation of estrogen [46]. This may also contribute to the lack of significant health benefits of flavonoid intake in women. Although further research is necessary to elucidate the precise mechanisms.

The RCS analysis revealed a nonlinear dose-response relationship, possibly U-shaped, between flavonoid intake and the risk of asthma in the overall population. The analysis also identified 1500 mg/day as the tolerable upper intake level for dietary flavonoids. Safe intake doses of flavonoids as dietary supplements have been studied [47]. The recommendations for safe dose ranges of dietary flavonoids vary from different countries. The U.S. Food and Drug Administration (FDA) has deemed high-purity quercetin as Generally Recognized as Safe (GRAS) under the intended conditions of use, with a daily intake of up to 1,250 mg considered safe [48, 49]. Italian regulations limit the maximum daily intake of mixed, unspecified flavonoids to 1,000 mg [50], while in Canada, the daily dose of quercetin as a natural health product ingredient is restricted to 1200 mg [51]. In light of our study findings, it is suggested that dietary flavonoid intake may play a role in respiratory health, with the tolerable upper intake level likely falling within the range of 1500 mg/d for the entire U.S. population.

We observed that the combined healthy effects of total flavonoids were particularly noteworthy in the WQS-asthma model and the emphysema model. Among the 29 flavonoids examined, Glycitein exhibited the highest health contribution of 26.2% in the WQS-emphysema model. Furthermore, Eriodictyol demonstrated the highest health contribution of 32.13% in the WQS-asthma model. Notably, Eriodictyol played a significant role in both emphysema and asthma. Glycitein, a soy isoflavone, possesses antioxidant, anti-inflammatory, and anticancer properties [52]. Kim, J. H et al discovered that a flavonoid related to Glycitein effectively inhibits the inflammatory pathway-induced mucus hypersecretion in human lung mucous epidermoid cells [53]. Eriodictyol, commonly present in citrus fruits, is a flavanone compound known for its numerous health benefits, including antioxidant, anti-inflammatory, hypolipidemic, and hypocholesterolemic properties [54]. Some studies have consistently reported the inhibitory effects of Eriodictyol on acute lung injury, which is precipitated by either inflammation or bacterial infection. These findings underscore the potential of Eriodictyol as a therapeutic agent in mitigating the severity of lung injuries associated with these conditions [55, 56]. Furthermore, a Japanese population study revealed a correlation between high plasma concentrations of Eriodictyol and a reduced risk of lung cancer [57]. These findings are congruent with our own, reinforcing the collective understanding of how dietary flavonoids can be leveraged to enhance respiratory health. The convergence of these results offers significant implications for the development of nutritional strategies aimed at reducing the incidence and severity of respiratory disorders.

The study possesses several advantages. Firstly, dietary flavonoid intake found to be associated with reduced risk of chronic respiratory disease, especially in the U.S. adult men. Secondly, A U-shaped dose-response relationship between dietary flavonoids and respiratory health was found, with 1500 mg/d being the tolerable upper intake level. Providing valuable information for understanding the optimal intake of flavonoids for U.S. adults. Furthermore, the study extensively examines the combined effects and contribution proportions of individual components within dietary flavonoids. It provides further direction for the application of dietary flavonoids in practical clinical applications.

This study also has certain limitations. First, the design of this study was based on a cross-sectional study, and it was not possible to clarify the causal association between dietary flavonoids and the risk of chronic respiratory disease seen. Second, the calculation of flavonoid intake was based on dietary type and corresponding flavonoid content, and the database did not have separate questionnaire data on flavonoid diets, so there was a corresponding recall bias for flavonoid intake. Finally, although corrections for relevant covariates have been made, there are still unknown confounders that can affect the interpretation of the results. More research is needed to validate the results when extrapolated beyond the U.S. population.

The results of this study first identified the existence of a possible protective effect of dietary flavonoid intake on respiratory health in men, contributing to the improvement of dietary management guidelines for clinical chronic respiratory disease populations. Moreover, this study found a possible U-shaped dose relationship between dietary flavonoids and the risk of respiratory disease, and gave recommendations on the maximum tolerable amount of dietary flavonoids, which could be useful for clinical dietary flavonoid intake guidelines.

## Conclusion

In the U.S. population, high dietary flavonoid intake may be associated with a low risk of chronic respiratory disease, and the association between flavonoid intake and chronic bronchitis and asthma was more significant in males The flavonoid components, Eriodictyol and Glycitein, contribute most significantly to respiratory health. The tolerable upper intake level of dietary flavonoids to respiratory health may be 1500 mg/day of U.S. adults.

## Supporting information

S1 Dataset (XLSX)
